# A Process Evaluation Protocol for Examining the Impact of Instructions for Correct Use of Child Car Seats Designed through a Consumer-Driven Process and Evaluated in a Field-Based Randomised Controlled Trial

**DOI:** 10.3390/ijerph17124508

**Published:** 2020-06-23

**Authors:** Julie Brown, Jane Elkington, Kate Hunter, Judith L. Charlton, Lynne E. Bilston, Andrew Hayen, Lisa Keay

**Affiliations:** 1 The George Institute for Global Health, University of New South Wales, Sydney 2042, Australia; j.elkington@neura.edu.au (J.E.); khunter@georgeinstitute.org.au (K.H.); l.keay@unsw.edu.au (L.K.); 2 Neuroscience Research Australia (NeuRA) and Faculty of Medicine, University of New South Wales, Sydney 2031, Australia; l.bilston@neura.edu.au; 3 Monash University Accident Research Centre, Monash University, Melbourne 3800, Australia; judith.charlton@monash.edu.au; 4 School of Public Health, University of Technology Sydney, Sydney 2007, Australia; andrew.hayen@uts.edu.au; 5 School of Optometry and Vision Science, University of New South Wales, Sydney 2052, Australia

**Keywords:** child restraints, misuse, injury, behaviour theory, randomised trial

## Abstract

The incorrect use of child car seats is common, with significant negative effects on crash protection for child passengers. There is currently little evidence for effective, practical countermeasures for incorrect use. The provision of clear and comprehensible materials on correct use supplied with restraints at the point of sale could be highly cost-effective and achieve similar benefits to restraint-fitting services or hands-on training; however, routinely supplied instructions in their current form are frequently difficult to understand. We are conducting a randomised controlled trial of the consumer-driven redesign of instructional materials, consisting of an instruction sheet, swing tags and online training videos. This paper presents the protocol that will be used in an innovate process evaluation that will use the primary outcome of overall serious misuse assessed at six months, together with a survey and semi-structured interviews to determine fidelity, dose and outcomes for all intervention participants. The study will assess intervention delivery and external factors that may impact the effectiveness of the intervention, including experience, health literacy, confidence and attitudes. When it has been conducted, this process evaluation will provide enhanced understanding of the mechanisms through which the intervention works or not, aspects of the implementation process key to success of the intervention and insight into how external factors influence the success of the intervention.

## 1. Introduction

The incorrect use of child car seats is a long-standing and widespread problem. In Australia, the latest available estimates are that one in two children have errors in the way they are restrained when travelling in cars [1]. Similar rates of incorrect restraint use are reported in Europe and North America [2,3,4,5]. There is a need to find effective interventions to reduce the rate of incorrect use given the significant negative effect that the incorrect use of child car seats has on crash protection for child passengers [6]. We previously estimated that for every 15% increase in correct use, we can expect a 10% reduction in serious injury and death among children involved in car crashes [6].

Through previous research, we have demonstrated that the use of restraint-fitting services is associated with a reduced likelihood of errors in restraint use [7]. However, at the population level, the use of restraint-fitting services remains relatively low [7], and large-scale one-on-one interventions are prohibitively expensive and resource intensive. Clear and comprehensible materials containing information on how to correctly use restraints supplied with the restraints at the point of sale could be highly cost-effective if this could achieve a similar benefit to hands-on assistance or training. Emerging work in this area has demonstrated that hands-on assistance and/or training in-person [8,9] via video [10] or through remote virtual assistance technology [11] is promising.

Worldwide, instructions are routinely supplied with child restraints by manufacturers. Increasingly, manufacturers are supplementing written instruction booklets and labels fixed to the restraints with videos. However, the continuing high rates of errors in use suggest that in their current form, these materials are not successful in reducing errors. The comprehension of current written instructions is likely problematic given evidence that child restraint product information is written at a reading level too high for most users [12]. This was further supported by Tsai and Perel [13], who explicitly identified the difficulties users had in interpreting instruction manuals when using them to install child seats under observation. These comprehension difficulties may explain the counterintuitive finding of one North American study that reported a higher likelihood of incorrect use among children of those reporting the use of instruction materials than those reporting non-use [14].

A small number of studies have examined the potential of redesigned information supplied with child restraints as a measure to reduce errors in use [15,16,17,18]. Two of these have focused solely on the design of labels fixed to the restraint [16,17], one focused on written instructions and labels fixed to the restraint [15], and most recently, we developed an enhanced set of materials incorporating written instructions, labels and video [18]. Generally, these studies have found improvement in some areas of correct child restraint use; however, only the work by Kramer et al. [17] and Hall et al. [18] achieved significant effects of re-design over current information on correct restraint use in laboratory-based trials. One key difference in the design process between those demonstrating some significant effect on correct use and those that did not was the inclusion of an iterative consumer-driven design step in the process of developing materials. The positive impact of including consumers in the design process of instructional materials aligns with the gold standard model for the user-centred design of health information materials developed by Sless and Wiseman [19]. This model was developed to ensure the comprehension and usability of materials and has been demonstrated to produce effective materials across several health disciplines [20,21].

In our recent work [18], we used the Sless and Wisemen model [19] to develop a set of materials, which we evaluated in a randomised controlled pilot trial in the laboratory. We found a significantly higher level of comprehension of key information related to correct use among those exposed to the prototype material than those exposed only to existing materials, and a significant linear relationship between the comprehension of this information and correct use of the restraint. Using a stringent, realistic measure of errors likely to have a significant deleterious effect on crash protection, we demonstrated a 27% reduction in errors among those exposed to the prototype compared to those exposed to existing materials [18]. While this impact on correct use is promising, it was achieved under controlled laboratory conditions with a participant population not entirely representative of child restraint users. To confirm the effectiveness of these prototype materials, we are now conducting a pragmatic field-based randomised controlled trial measuring impact on correct use six months after the purchase of a child restraint system.

To be effective as a countermeasure to errors in use in the real world, the aim of the prototype materials is to elicit a specific behaviour: the correct installation and use of a child restraint. According to the integrated model of behaviour theory [22], a person will likely adopt a behaviour if they have a strong intention to perform the behaviour, have the necessary skills and abilities to perform the behaviour, and there are no constraints preventing behavioural performance. As noted above, our consumer-driven design of materials is associated with increased levels of comprehension compared to existing instructions and therefore likely targets the “skills and abilities” component of this model of behaviour. In theory, in a randomised controlled trial, the only difference between the groups should be exposure to the intervention, and therefore, aspects related to the “intention” and “physical constraints” components of the behaviour model should be balanced between the groups. However, unlike in the controlled laboratory environment, in the real world, there may be differences in how participants access and respond to the materials and other factors that may moderate the effectiveness of the intervention. Understanding these factors is key to the successful translation of the prototypes to a broad-based measure to reduce errors in use if the intervention is found to be effective, or to facilitate improvements to the intervention if it is found to not reduce errors in use in the real world and over time.

Process evaluation is a common tool used in pragmatic trials to assess the impact of intervention delivery and access within the trial, as well as to gain understanding about how and why the intervention did or did not have its desired impact [23,24], and publishing protocols and analysis plans prior to the conducting of the process evaluation, as is routine in randomised controlled trials, is becoming increasingly common.

In this paper, we present the protocol for our planned process evaluation within a pragmatic randomised controlled trial. The specific aims of our planned process evaluation are to examine the impact of intervention delivery and external factors potentially altering the intervention impact on the relationship between intervention exposure and the correct use of the restraint six months post-purchase.

## 2. Materials and Methods

In our randomised controlled trial, we aim to recruit 440 people who have decided to purchase one of two different commercially available child restraints. The restraints include a rearward/forward facing convertible car seat (suitable for children aged 0 to approximately 4 years) and a convertible forward facing/booster seat restraint (suitable for children from 4 years to approximately 8 years). Both are designed to be installed in a vehicle using the vehicle seatbelt and a top tether. Neither includes the means for attachment to ISOFIX connectors in a vehicle. The rationale for not including ISOFIX-compatible restraints is that while the mandatory Australian product standard (AS/NZS 1754) allows for ISOFIX-compatible restraints, it requires that all child restraint systems (including those that are ISOFIX compatible) are able to be anchored to the vehicle by the seat belt and top tether anchorage system. This, together with the fact that the uptake of ISOFIX-compatible restraints is still relatively low, means the seatbelt/top tether anchorage system is currently the dominant anchorage system in Australia. The specific makes and model of restraints used in this study were arbitrarily chosen during the intervention material design process. The recruitment strategies encompass a broad-brush approach to reaching people who may soon be purchasing a new child restraint. These strategies include posters and postcards in the ante-natal areas of hospitals, child care centres and pre-schools; the use of social media such as parenting forums and Facebook groups; and the websites of organisations that may be visited by parents and expectant parents. On recruitment, participants are randomly allocated to the control group (*n* = 220) or the intervention group (*n* = 220), and control or intervention materials are delivered to the participants with the purchased restraint along with the usual materials supplied with the restraint. The study materials are therefore supplementary to the usual packaging, instructions and labels supplied with the restraint, as required by the mandatory Australian product standard (AS/NZS 1754). The intervention group receive the set of materials we developed using the consumer-driven design process and previously shown to be effective in a laboratory trial [18]. The materials delivered to the control group consist of a generic child passenger safety postcard describing the legal requirements for child restraint use in New South Wales, Australia. Participants are reimbursed AU$100 for their involvement in the study. As we aim to examine the impact of implementation and mechanisms underpinning the effect of the intervention, this process evaluation will only include data collected from participants within the intervention group of our pragmatic field trial. The process evaluation will be undertaken blinded to trial outcomes as recommended by MRC guidelines [23].

### 2.1. The Intervention and Randomised Controlled Trial

The intervention materials consist of an A3 size instruction sheet, a set of four swing tags designed to be fixed to the restraint and an online video demonstrating the overall instructions and use process, as well as separate video snippets relevant to key tasks. The video is accessible via QR codes printed on the A3 instruction sheet and on the swing tags. The web address to access the video is also included on the written materials. The design process involved developing an initial draft set of materials whose content and layout were informed using procedural task analysis; a review of human factors, instructional design literature and previous studies [15,16,17]; and a series of focus groups with users [25]. These were then refined through iterative user-testing (Hall et al., unpublished data) following the Sless and Wiseman model [19], which involved groups of the target population being exposed to the materials, with the subsequent refinement of the materials until a pre-defined criterion of a group average of 80% correct use and 80% correct comprehension was achieved.

The final prototype intervention materials target both the correct installation of the restraint within the vehicle and correct securing of the child within the restraint. An A3 instruction sheet and an online video detail the overall instructions and use process. The swings tags show what should be checked every trip and are designed to prompt users to check for the most common errors, checking the seat belt attachment (which anchors the child car seat to the car), the top tether strap attachment, and that the child is secured properly within the harness.

Prior to being sent their child restraint, participants are told over the phone that the study materials will be inside the box in which the child restraint is packaged. They are instructed to look out for the zip-lock bag with the study materials inside. To keep participants “blinded” to their group allocation, they are informed that there are two sets of information materials, that each is technically correct and approved by child safety experts, and that participants will be randomly assigned to one or the other. The intervention materials come with a printed set of instructions recommending that the A3 instruction sheet be kept, folded, in the pouch on the child car seat provided for the manufacturer’s instruction booklet and that the swing tags be adhered to the plastic shell of the car seat, towards its rear, using the adhesive attachment provided. As part of the participant information statement, participants are told that they do not need to do anything differently than they are inclined to do, in terms of the installation of and use of the child restraint and the use of any informational materials about child restraints.

Once a participant is enrolled in the study (i.e., has purchased the car seat), they are assigned the next-in-line participant identification number, which has a pre-assigned random group allocation: intervention or control. A researcher gathers the relevant study materials and checks, using a QR code reader, that the QR code link is working properly on every third set of intervention group materials. The study materials are then placed in a transparent zip-lock bag. The zip-lock bag is inserted into the top of the box of the purchased car seat, through the open slit of the cardboard flaps. The box does not need to be opened to do this. The address label for the courier is then taped to the box, and the courier is contacted for the pick-up of the car seat and delivery to the participant.

The primary outcome measure is assessed during a home visit arranged for six months after the participant commences use of the child restraint. The primary outcome is overall serious misuse of the restraint as observed by a trained researcher during the home visit. Errors are recorded using an established pro-forma [1] and categorised as minor or serious using the same definitions we have used previously [18]. Our dichotomous primary outcome variable is therefore categorised as “overall serious misuse” where there is at least one serious error, or two or more minor errors, and “no serious error” when there is one minor error or no errors. A survey will also be delivered to the participant during the home visit.

### 2.2. Theoretical Framework for the Process Evaluation

**Figure 1:**
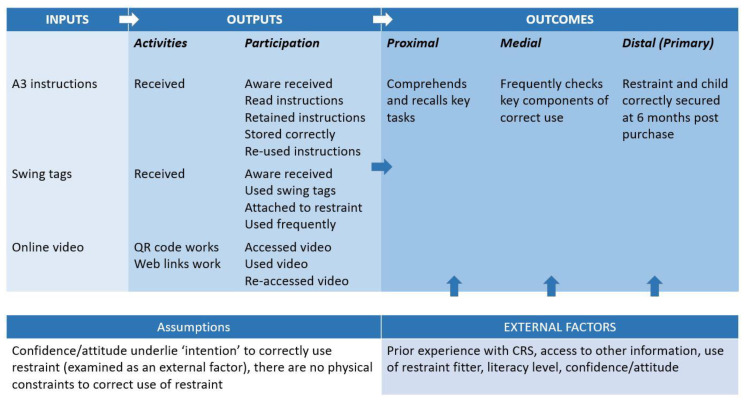
Logic model describing the hypothesised relationship between the intervention being tested and the primary trial outcome, the correct use of the restraint at six months post-purchase.

As noted above, we believe the intervention materials will target the “skills and knowledge” component of the integrated model of behavior [22] and hypothesise that the intervention will increase the correct use of the restraint at six months post-purchase as depicted in the simple logic model illustrated in Figure 1.

The logic model depicted in Figure 1 will provide a framework for the process evaluation. As noted in Figure 1, we have assumed there are no physical constraints barring correct use among the study participants and that differences among study participants in their intention to correctly use the restraint can be assessed by measuring their confidence in and attitudes towards correct use. We therefore have defined confidence and attitudes to correct use as one of the external factors potentially impacting the relationship between the intervention and correct use. Other external factors being considered are level of prior experience using child car seats, access to other information about the correct use of car seats, the use of a professional restraint fitter, and the participants’ health literacy level.

Our hypothesised pathway between the intervention and the desired outcome requires the delivery of the intervention materials as planned (fidelity), that the participants receive each of the intended materials (dose delivered) and that participants access and use the materials (dose received). In the short term, we hypothesise that if the implementation of the intervention is successful, the participant will comprehend the intervention materials (which we have tested previously in a laboratory trial [18]) and recall key tasks about using a car seat correctly. This will lead the participant to continue to check key aspects of correct use, and ultimately, the car seat will continue to be correctly used six months after purchase.

### 2.3. Process Measures

Table 1 summarises the planned process and outcome measures, data collection methods, and links to our evaluation framework depicted in Figure 1.

The reach of our intervention is fixed, with all participants expected to receive the same intervention components. The fidelity and dose delivered are also constant due to delivery of the materials with the purchased restraint and our processes for checking that the QR codes linking to online videos are continuing to work. As shown in Table 1, the bulk of the process evaluation measures are related to the dose received, and these will also be obtained through the semi-structured interview delivered at the six-month follow-up and researcher observation. Full details of the questions asked and construction of the dose received variables are provided in Supplementary Materials.

As detailed in Table 1 and Supplementary Materials, there are five measures for participation related to awareness and the self-reported use of the A3 instructions. An A3 instruction participation score will be calculated by summing the dichotomous codes (0 or 1) for each individual measure to deliver a score out of 5. For the swing tags, there are four individual measures, and for the video, there are three separate measures. The participation scores for each of these different components of participation will be calculated in the same way as described for the A3 instruction sheet.

Figure 1 defines the set of external factors we hypothesise may impact the effectiveness of the intervention, and as shown in Supplementary Materials, measures to assess these external factors will primarily be collected through the semi-structured interview during the follow up visit. Prior experience with child restraint systems will be gauged by asking about other children and the participants’ self-reported previous experience of installing and using child restraints, use of restraint fitters and other sources of information. Health literacy will be objectively measured using the Single Item Literacy Screener (SILS), “How often do you need to have someone help you when you read instructions, pamphlets, or other written material from your doctor or pharmacy [26]?”. This has previously been found to be a valid, reliable and efficient means for establishing health literacy [27]. We have included the same Likert scale item we have previously used to assess this parental characteristic [28,29]. The full details of the questions used to elicit responses used to measure external factors are also provided in Supplementary Materials.

### 2.4. Outcomes

Proximal, medial and distal (primary) outcomes are summarised in the logic model shown in Figure 1, and the measures are summarised in Table 1. Our distal outcome is the same as the primary outcome being used and described above and is being objectively assessed by researchers during the six-month home visit inspection of the restraint. Our proximal and medial outcomes will be obtained through the survey with the parent at the same home visit. Further details of the questions used to elicit responses used to make these are assessments are provided in Supplementary Materials.

### 2.5. Analysis

This process evaluation is designed as a quantitative evaluation of the intervention process and hypothesised relationship between the intervention and correct use of the restraint as depicted in Figure 1.

Descriptive statistics will be used to report the fidelity, dose delivered and dose received, as well as to describe the sample composition by external factors. Logistic regression will then be used to test the relationship between the intervention and correct use, accounting for the impact of intervention delivery and external factors. Structural equation modelling will be used to test the logic model through the intermediary outcomes as depicted in Figure 1.

### 2.6. Sample Size

Our pragmatic field-based randomised controlled trial to test the effectiveness of the materials we developed through a consumer-driven approach requires 440 participants (220 in each group) to detect a 15% difference in correct use between the intervention and control groups with 80% power at the 5% level allowing for 20% drop out. Process evaluation data will be available for 176 intervention participants so will be sufficient to quantify differences in dose received scores, and the modelling of the relationship between the intervention and correct use taking into account the impact of intervention delivery and external factors.

## 3. Discussion

This process evaluation protocol has been developed to gain understanding of the impact of intervention delivery and external factors on the impact of our consumer-driven materials on the correct use of restraints six months after restraint installation. This will be the first time that process evaluation grounded on a behavioural change theoretical framework will be applied in the context of the correct use of child restraints. It will provide enhanced understanding of the mechanisms through which the intervention works or not, aspects of the implementation process that are key to the success or otherwise of the intervention, and insight into how external factors influence the success or otherwise of the intervention.

In previous work, 90% of Australian parents have self-reported the use of instructions supplied with restraints [30], but there is real possibility that a null finding in our randomised controlled trial could be influenced by parents not using the materials supplied. Knowing this would be very important to future efforts to intervene to counter the incorrect use at the point of purchase. Similarly, child restraint users span the spectrum of demographics in terms of literacy, experience with child restraints and the use of other resources, or not, to assist in the correct use of child restraints. It is possible that these factors might influence the effectiveness of our intervention. Understanding the impact of these on factors on our trial outcome is critical to the future implementation of this intervention as well as for future developments. Importantly, this process evaluation is grounded on a theoretical framework, which provides a mechanism to explore how the intervention works or the critical actions along the path of being exposed to instructions on how to use child restraints to the desired end-point of the ongoing correct use of child restraints. This will also provide invaluable information for the development of future measures to counter the incorrect use of child restraints.

The potential limitations of this study include the assumption of 100% fidelity and dose received. This assumption is made on the intervention delivery protocol that requires checks that materials are packaged as intended, and the QR code and web link checks indicate participants will be able to access the video. There is, however, some possibility that something unexpected happens between when the restraint is picked up by the courier and when it is opened by the participant. Most of the process measures and interim outcomes (proximal and medial) are collected through self-reporting. Therefore, there is potential for social desirability bias. However, in earlier work, we found some evidence for the good reliability of self-report measures related to the correct use of child restraints [31]. Finally, working within the framework of the integrated behaviour theory model, we have assumed (see Figure 1) that there are no physical constraints limiting the participants’ opportunity to correctly use the restraint. Data necessary to test this assumption are not being collected during the follow up visit. If the intervention fails to improve correct use in the intervention group compared to the control group, and this process evaluation fails to identify any factors influencing that outcome, this is an area where further study would be warranted.

The strength of this study will be the ability to systematically explore factors underpinning the outcome of the randomised controlled trial and the mechanisms through which the intervention works or fails to address the incorrect use of child restraints.

## 4. Conclusions

The incorrect use of child car seats is a common problem that may be reduced through enhanced instructional material. This process evaluation of our randomised controlled trial of additional instructional material for installation and securing the child will systematically evaluate intervention delivery and external factors that may impact the effectiveness of the intervention, grounded on a behavioural change theoretical framework. It will provide enhanced understanding of the mechanisms through which the intervention works or not, aspects of the implementation process key to the success or otherwise of the intervention, and insight into how external factors influence the success or otherwise of the intervention.

## Figures and Tables

**Table 1:** Process measures.

Component/Logic Model Link	Measures	Method	Data Type
Fidelity, Dose Delivered/Output Activities	A3 instructions & swing tags received, QR codes & web links work	Assumed 100%	N/A
Dose received/Output Participation	A3 instruction sheet participation score (aware received A3 instructions, read instructions, retained instructions, stored correctly, re-used instructions); swing tag participation score (aware received swing tags, used swing tags, swing tags attached to restraint, swing tags used frequently); video participation score (accessed video, used video, re-accessed video)	Survey questions	Continuous
Proximal Outcome	Comprehends and recalls key tasks	Survey question	Categorical
Medial Outcome	Did the participant continue to check key components of correct use?	Survey question	Categorical
Distal (Primary) Outcome	Was the restraint used correctly at six- month visit?	Home visit check	Categorical
External Factors	Health literacy level, prior experience with car seats, access to correct use information from other sources, use of a restraint fitter, confidence/attitude	Single item health literacy, survey questions	Health literacy continuous/all others categorical

## Supplementary Materials

The following are available online at https://www.mdpi.com/1660-4601/17/12/4508/s1, Table S1: Full details of survey and semi-structured interview questions, and construction of dose received variables.
