# Supplementary material for: A Process Evaluation Protocol for Examining the Impact of Instructions for Correct Use of Child Car Seats Designed through a Consumer-Driven Process and Evaluated in a Field-Based Randomised Controlled Trial
Source: Int J Environ Res Public Health. 2020 Jun 23;17(12):4508. doi: 10.3390/ijerph17124508 (PMC7345236; doi:10.3390/ijerph17124508)
Supplement: Supplementary file 1 [file ijerph-17-04508-s001.pdf]

Table 1. Full details of survey and semi-structured interview questions, and construction of dose received variables.

Process Component	Measure	Survey question(s)	Response Options	Code	Process Measure	Process Score
Dose received/ Output Participation	1. Aware received A3 instructions	Do you recall the informational material which was included in the car seat box when it was sent to you by the research team approximately 6 months ago?	No, Yes (If yes, please list the material you recall being sent)	0 = No or no mention of A3 sheet<br>1= Yes & mentions A3 sheet	A3 Instruction Sheet Participation Score	Sum Measure 1-5 Code to deliver score/5
	2. Read Instructions	Have you, or your partner, read or referred to any of the following informational material about the installation and use of the car seat since you received it? (Tick all that apply – leave blank if the answer is "no"). ….An A3 colour guide	Self -Yes DK;<br>Partner - Yes DK<br>(leave blank if "no")	0=DK/No<br>1= Self/Partner		
	3. Retained instructions	Of the study materials you received, where did you keep these?<br>….A3 colour guide	Lost it/didn't keep it<br>Pocket in car seat<br>Glovebox<br>Kept it somewhere else	0= Lost it/didn't keep it<br>1= anything else		
	4. Stored correctly	Observed by Researcher	No<br>Yes	0 = A3 not stored correctly<br>1= stored correctly		
	5. Re-used instructions	Which of the following best describes what you did with this material?:	Nothing<br>Read it once<br>Read it a few times<br>Read or referred to it multiple times	0 = 0 or 1<br>1 = 2 or 3		

	6. Aware received swing tags	Do you recall the informational material which was included in the car seat box when it was sent by the NeuRA research team approximately 6 months ago?	No,<br>Yes (If yes, please list the material you recall being sent)	0 = no mention of swing tag<br>1= mentions swing tag	Swing Tag Participation Score	Sum Measure 6-9 Code to deliver score/4
	7. Used Swing tags	Have you, or your partner, read or referred to any of the following informational material about the installation and use of the car seat since you received it? (Tick all that apply – leave blank if the answer is "no").<br>….Swing tags	Self -Yes DK;<br>Partner - Yes DK<br>(leave blank if "no")	0=DK/No<br>1= Self/Partner		
	8. Swing tags attached to the restraint	Observed by Researcher	No<br>Yes	0 = swing tags not attached<br>1= swing tags attached		
	9. Swing tags used frequently	(If yes) Which of the following best describes what you did with this material?:	Nothing<br>Read it once<br>Read it a few times<br>Read or referred to it multiple times	0 = 0 or 1<br>1 = 2 or 3		
	10.Video Accessed	Have you, or your partner, read or referred to any of the following informational material about the installation and use of the car seat since you received it? (Tick all that apply – leave blank if the answer is "no"). ….QR Code Video	Self -Yes DK;<br>Partner - Yes DK<br>(leave blank if "no")	0=DK/No<br>1= Self/Partner	Video Participation Score	Sum Measure 10-12 Code to deliver score/3
	11. Used video	(If yes) Which of the following best describes what you did with this material?:	Nothing<br>Read it once<br>Read it a few times	0= Nothing<br>1= Anything else		

			Read or referred to it multiple times			
	12. Re-accessed video	(As above)		0= Nothing or read once or a few times<br>1= Read or referred to it multiple times		
Proximal outcome	Comprehends & recalls key tasks	What do you think are the important things to check on your restraint before driving on every trip?<br>*Please circle the important features to check for in the figure of the child restraint you are using (forward facing <u>or</u> rear-ward facing) and briefly describe what you are checking for. Alternatively, you may show and describe to the researchers the features on your child restraint which you should check*	Diagram of restraint	0= Completely incorrect<br>1=Partially correct<br>2=Correct		
Medial outcome	Did the participant continue to check key components of correct use?	Q9. Since its initial installation in your motor vehicle, how often has the installation been checked by anyone?	It has not been checked since then **(Go to Q10)**<br>Less than once a month<br>Around every 3-4 weeks<br>Around every 1-2 weeks<br>More than once a week	0=it has not been checked<br>1= Less than once a month<br>2= Around every 3-4 weeks<br>3=Around every 1-2 weeks<br>4=More than once a week		
		List what is generally checked	(open-ended response)<br>(Interviewer can document response if	0=Lists no key tasks<br>1=Lists some key tasks		

			respondent points to things without knowing their names.)	2= List all key tasks		
	Frequently checks key components of correct use	CONSTRUCTED FROM ABOVE	Does not check frequently<br>Checks frequently (more than once a week) but no key tasks<br>Checks frequently but not all keys tasks<br>Checks frequently and knows keys tasks	0,1,2,or 3=0<br>4+0=1<br>4+1=2<br>4=2=3		
Distal (Primary) outcome	Was the restraint used correctly at 6 month's visit?	Observed by Researcher	Definition of serious misuse	0=No serious misuse<br>1= serious misuse		
External factor	Health literacy level	How often do you need to have someone help you when you read instructions, pamphlets, or other written material from your doctor or pharmacy?	Always<br>Often<br>Sometimes<br>Rarely<br>Never	Always= 5<br>Often =4<br>Sometimes =3<br>Rarely =2<br>Never =1		
	Prior experience with car seats	**Prior** to first using the child car seat that you purchased that is part of this study, which of the following applies to your experience with installing child car seats? ***(tick all that apply)***	I had no prior experience with child car seats<br>My partner had no prior experience with child car seats	0= self and partner no prior experience<br>1= self or partner had prior experience		

			I had installed child car seat/s before (# times)<br>My partner had installed child car seat/s (# times)	2= self and partner had no prior experience		
	Access to other information	Other information ticked something other than manufacturers instructions described	0= No<br>1=Yes			
	Use of restraint fitter	Mentions restraint fitter in response to relevant questions	0=No<br>1=Yes			
	Confidence/ attitude	I know everything I need to know to ensure my child is properly restrained in a car	Strongly agree<br>Agree<br>Undecided<br>Disagree<br>Strongly disagree	Stongly agree and agree = confident = 1<br>All others – Not Confident = 0		
